# Resistance to second generation antiandrogens in prostate cancer: pathways and mechanisms

**DOI:** 10.20517/cdr.2020.45

**Published:** 2020-09-17

**Authors:** Shiv Verma, Kumari Sunita Prajapati, Prem Prakash Kushwaha, Mohd Shuaib, Atul Kumar Singh, Shashank Kumar, Sanjay Gupta

**Affiliations:** ^1^Department of Urology, Case Western Reserve University, Cleveland, OH 44106, USA.; ^2^The Urology Institute, University Hospitals Cleveland Medical Center, Cleveland, OH 44106, USA.; ^3^School of Basic and Applied Sciences, Department of Biochemistry and Microbial Sciences, Central University of Punjab, Bathinda 151001, India.; ^4^Department of Nutrition, Case Western Reserve University, Cleveland, OH 44106, USA.; ^5^Divison of General Medical Sciences, Case Comprehensive Cancer Center, Cleveland, OH 44106, USA.; ^6^Department of Urology, Louis Stokes Cleveland Veterans Affairs Medical Center, Cleveland, OH 44106, USA.

**Keywords:** Prostate cancer, second-generation antiandrogens, androgen receptor, castration resistance prostate cancer

## Abstract

Androgen deprivation therapy targeting the androgens/androgen receptor (AR) signaling continues to be the mainstay treatment of advanced-stage prostate cancer. The use of second-generation antiandrogens, such as abiraterone acetate and enzalutamide, has improved the survival of prostate cancer patients; however, a majority of these patients progress to castration-resistant prostate cancer (CRPC). The mechanisms of resistance to antiandrogen treatments are complex, including specific mutations, alternative splicing, and amplification of oncogenic proteins resulting in dysregulation of various signaling pathways. In this review, we focus on the major mechanisms of acquired resistance to second generation antiandrogens, including AR-dependent and AR-independent resistance mechanisms as well as other resistance mechanisms leading to CRPC emergence. Evolving knowledge of resistance mechanisms to AR targeted treatments will lead to additional research on designing more effective therapies for advanced-stage prostate cancer.

## Introduction

Prostate cancer remains the most commonly diagnosed malignancy and the second leading cause of cancer related deaths among men in the United States^[1]^. According to an estimate by the American Cancer Society, approximately 191,930 new cases of prostate cancer will be diagnosed and 33,330 deaths will occur from this disease in 2020^[2]^. The majority of deaths from prostate cancer is due to advanced-stage metastatic spread dependent on the androgen receptor (AR). Because of the crucial role of AR in the development and progression of prostate cancer, androgen deprivation therapy (ADT) is the standard-of-care therapy for relapsed or metastatic patients attained by either surgery or medical castration with luteinizing hormone-releasing hormone (LHRH) agonists or antagonists^[3]^. The first-generation antiandrogens such as hydroxyflutamide and bicalutamide have been used over many years for the treatment of prostate cancer. Most tumors initially shrink in response to ADT, but eventually emerges as castration-resistant prostate cancer (CRPC) within 18-24 months. Detailed studies on CRPC have shown their dependency on the AR signaling axis despite systemic depletion of androgens by various mechanisms. Upon progression, CRPC eventually metastasizes to the bone and later to other distant organs. Patients with metastatic CRPC (mCRPC) exhibit poor prognosis and low predicted overall survival of less than 2 years from the initial time of progression; such patients account for a large portion of the prostate cancer-related deaths. Two novel agents, abiraterone acetate and enzalutamide, were developed as second-generation AR axis-targets agents that offer more effective inhibition of the AR pathway^[4]^. Understanding the mechanisms of resistance to the second-generation antiandrogens is the key to develop more effective therapies. In this review, we will discuss the current knowledge regarding the pathways leading to castration resistance and the mechanisms of resistance against these agents.

## AR

**Figure 1:**
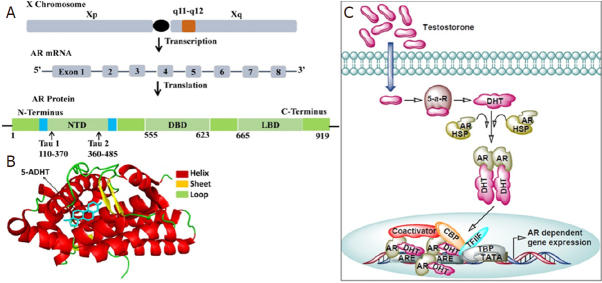
The androgen-androgen receptor signaling pathway. A: The androgen receptor gene resides on the long arm of X chromosome (locus Xq11-Xq12). Upon transcription it produces mRNA containing 8 exons interrupted by introns which codes for the AR protein made up of 919 amino acids. AR protein contains several functional domains such as N-terminal domain (NTD), DNA binding domain (DBD) and ligand binding domain (LBD); B: Ligand binding domain of androgen receptor in complex with its ligand 5-α-dihydrotestosterone (5ADHT). The crystal structure of the androgen receptor ligand binding domain in complex with 5-alpha dihydrotestosterone (PDB ID 1T7T with resolution 1.70Å) was downloaded from RCSB protein databank. The PyMOL molecular visualization system was used to represent the protein-ligand complex in cartoon-sticks form; C: General mechanism of AR signaling. Testosterone diffuses into the cells and gets converted into dihydrotestosterone (DHT) via the action 5-α-reductase (5-a-R). DHT binds to the ligand binding pocket of androgen receptor (AR) and promotes its dissociation from the heat shock protein (HSP). Free AR then translocate into the nucleus and binds to androgen receptor element (ARE) present in the promoter region of AR responsive genes. At the promoter AR recruits components of basal transcriptional machinery such as TATA binding protein (TBP), transcription factor IIF (TFIIF), and cAMP responsive element binding protein (CRBP) which ensures the transcription of AR responsive genes

AR play an essential role in the development and function of a normal prostate gland in men. Aberrant AR expression is primarily involved in prostate carcinogenesis that are AR sensitive, but is also engaged in the metastatic growth of CRPC, in fact, the androgen axis continues to play an important role in the function and growth of CRPC^[5,6]^. AR is a nuclear ligand-activated transcriptional factor, a member of the steroid hormone nuclear receptor family, whose coding gene is located on the X chromosome at the locus Xq11-Xq12. AR consists of three main functional domains, the N-terminal transactivation domain (NTD) comprising of exon 1, a central DNA-binding domain (DBD) constitutes exons 2-3, and a C-terminal ligand-binding domain (LBD) spanning between exons 4-8 [Figure 1A and B]^[7]^.

The presence of a hinge region between the DBD and LBD is involved in nuclear localization and degradation. The N-terminus has a unique LxxLL-like motif, which binds to a hydrophobic cleft of the C-terminus generated by ligand binding to the receptor. This binding stabilizes the ligand binding caused by physical interactions between the N-terminal and C-terminal of the receptor^[8]^. The initial N-C interaction occurs in the cytoplasm. AR binds with androgens testosterone and dihydrotestosterone. Of note, adrenal androgens have lower binding affinity with the AR. Upon binding of AR with androgens, the complex acts as a transcriptional activator. In the absence of androgens, heat shock protein (HSP) binds to AR, therefore, AR remains inactive in the cytoplasm. Binding of androgens with AR induces a conformational change, resulting in the dissociation of HSP from AR, and the AR dimer translocate to the nucleus where it binds to androgen responsive elements of genomic DNA and regulate several target genes involved in growth and proliferation [Figure 1C]^[9,10]^.

## AR targeting agents

Several reports suggest that both AR responsive or refractory prostate cancer harbor increased AR expression due to genetic amplification of AR genes and AR enhancer elements^[11-14]^. Hence, research on prostate cancer treatments have targeted agents involved in the androgen receptor signaling axis. Androgen deprivation therapy (ADT), which inhibits transcriptional activity of AR, is utilized in the treatment of advanced prostate cancer. The therapeutic strategies involved in ADT strive to reduce levels of circulating androgen by surgical or chemical castration, and use antiandrogen to prevent androgen from binding to the AR. Circulating androgen levels are reduced by more than 90% within 24 h of surgical castration^[15]^. Chemical castration could be performed by using luteinizing hormone-releasing hormone (LHRH) agonist such as leuprolide acetate and goserelin acetate or with LHRH antagonist including degarelix. The first-generation nonsteroidal antiandrogens that are in clinical use include flutamide, nilutamide, and bicalutamide. Over past several years, these LHRH agonists and antiandrogens have been in the clinical use for the treatment of advanced-stage prostate cancer; however, this therapy is palliative and not curative. Various types of antiandrogens have been developed in past decades [Table 1]. These include antiandrogens such as enzalutamide, biculatamide, niculatamide, apalutamide, flutamide, cypreterone, abiraterone acetate, and darolutamide. Several of these antiandrogens have received FDA approval and are clinically prescribed for the treatment of advanced-stage prostate cancer. Enzalutamide, apalutamide, and darolutamide block AR based testosterone signaling in the cells by interrupting the interaction between androgens and ARs^[16]^, bicalutamide binds with AR ligand binding site, and flutamide compete with DHT for AR and inhibit the translocation of AR into nucleus, preventing AR-based downstream signaling^[17,18]^. Abiraterone acetate is a CYP17 enzyme inhibitor that inhibit the biosynthesis of testosterone^[19]^. The search for more effective therapies to block the transcriptional activity of AR remains the focus, of which the antiandrogen enzalutamide and the CYP17 enzyme inhibitor abiraterone acetate are discussed in detail.

**Table 1:** Various examples of antiandrogens agents*

Antiandrogens	Mode of action
Enzalutamide	AR antagonist
Bicalutamide	AR antagonist
Ostarine	Selective AR modulator
Apalutamide	Selective and competitive inhibitor
Galeterone	CYP17 inhibitor and AR antagonist
Flutamide	AR antagonist
Cyproterone Acetate	AR antagonist
AZD3514	AR down regulator
Spironolactone	AR Antagonist
Ligandrol	Selective AR modulator
Triptophenolide	Selective AR modulator
Testolone	Selective AR modulator
EPI-001	AR N-terminal domain antagonist
Darolutamide	AR antagonist and blocks AR nuclear translocation
Dehydroepiandrosterone	AR agonist
Nilutamide	AR antagonist

*Data was extracted from https://www.selleckchem.com/screening/fda-approved-drug-library.html

## Antiandrogens-abiraterone acetate and enzalutamide

Abiraterone acetate (Zytiga®) is a molecule whose structure is similar to pregnenolone. Abiraterone acetate selectively and irreversibly blocks the production of intratumoral androgen biosynthesis by inhibiting cytochrome P450 17A1 (CYP17A1), a cytochrome p450 enzyme required for the production of androgens through both 17α-hydroxylase and C17, 20-lyase activity^[20]^. These enzymes are also required for the synthesis of steroids including progesterone, glucocorticoids, mineralocorticoids, and estrogens^[21]^. The major function of CYP17A1 enzyme is to convert pregnenolone to dehydroepiandrosterone. CYP17A1 inhibition ultimately reduces the number of circulating androgens available to activate the AR^[22]^. Loss of CYP17A1 activity results in significant loss of androgen production, specifically in the peripheral organs, and loss of adrenal androgen production in particular^[23]^. Inhibition of CYP17A1 results in higher levels of urinary metabolite 3α5α-17HP which is correlated with the excretion of androsterone^[22]^. Overexpression or genomic changes in CYP17A1 contributes to abiraterone acetate resistance^[24]^. Using a xenograft mouse model, Chang *et al*.^[25]^ demonstrated that the HSD3B1 (1245C) mutation contributes to abiraterone acetate-resistant progression to CRPC, though the clinical significance is not completely elucidated. Abiraterone acetate has been shown to be 10-30 times more effective than ketoconazole, a non-specific inhibitor of p450 enzymes used to rapidly reduce androgen production^[23]^. Abiraterone acetate received FDA approval for the treatment of CRPC through COU-AA-301 and COU-AA-302 phase III clinical trials^[26,27]^. The double-blinded, placebo-controlled COU-AA-301 trial was conducted in chemotherapy-pretreated metastatic CRPC patients demonstrating 3.9-month median overall survival for patients treated with abiraterone acetate^[26]^. Initially, it remains an effective and essential paradigm in the treatment of metastatic CRPC, but AR reactivation drives prostate cancer to lethal CRPC phenotype in all patients despite effective testosterone suppression^[16]^.

Enzalutamide (MDV3100, Xtandi®) is another multi-targeted second-generation AR inhibitor impeding testosterone binding to AR, AR nuclear translocation, AR binding to DNA, and co-activator recruitment^[23]^. Enzalutamide was designed to overcome the limitations of first-generation agents such as bicalutamide or flutamide^[16]^. Enzalutamide exhibits three mechanisms of action: first, it prevents ligand binding and AR activation by binding to the AR LBD; second, it prevents AR translocation to the nucleus; and third, it inhibits the transcription of target genes by preventing binding of AR to DNA. FDA approval of enzalutamide is based on the AFFIRM and PREVAIL phase III clinical trials. AFFIRM was a double-blind, placebo-controlled trial that demonstrated a median overall survival of 4.8 months longer in patients with metastatic CRPC who were treated with enzalutamide. Additionally, PREVAIL was also a double-blind, placebo-controlled trial that revealed a 2.2 month longer median overall survival in chemotherapy-naïve metastatic CRPC patients treated with enzalutamide^[28,29]^.

## Cellular signaling pathways in antiandrogen resistance

Therapeutic agents in prostate cancer can develop resistance by primary or acquired mechanisms. Genetic changes, including amplification, mutation, or translocation of driver genes leading to AR splice variants and point mutations, are examples of processes causing enzalutamide or abiraterone acetate drug resistance^[16,30]^. Additionally, progression to AR independent forms of prostate cancer such as neuroendocrine prostate cancer is frequently observed post-enzalutamide treatment. This highlights a need to pursue development of novel strategies to target molecular mechanisms of castrate resistance. Despite the initial response of enzalutamide and abiraterone acetate in CRPC patients, the secondary resistance mechanisms unavoidably result in clinical progression of the disease. Thus, it is important to understand the mechanisms and pathways of resistance towards antiandrogens for which several preclinical models have been developed in order to investigate the underlying mechanisms.

**Figure 2:**
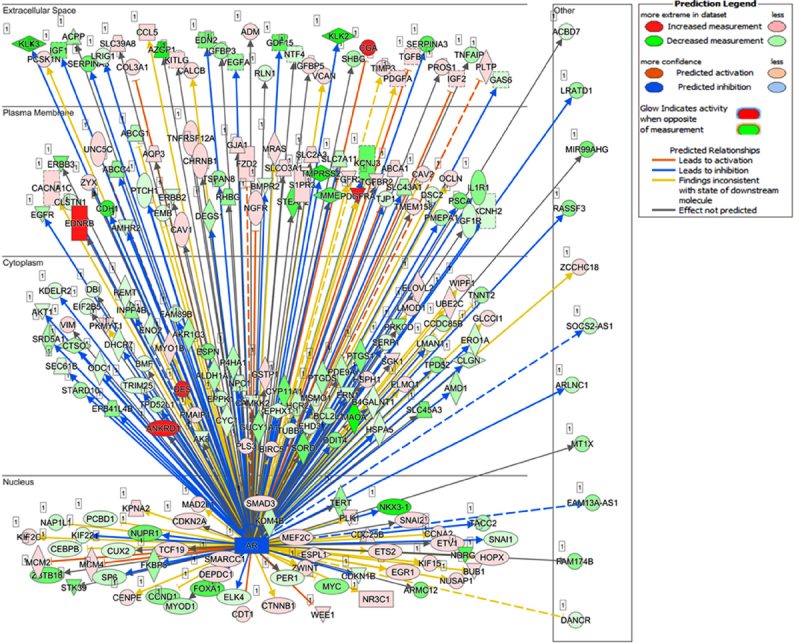
Distinct gene expression pattern of LNCaP-enzalutamide resistant cells in relation to AR. LNCaP cells treated with antiandrogen enzalutamide to generate LNCaP-enzalutamide resistant cells. These resistant cells show distinct gene expressed pattern in relation of AR in nucleus, cytoplasm, plasma membrane, and extracellular space. The red color shows increased expression, pink color shows less expression whereas green color shows decreased expression. The blue arrowhead shows genes leads to inhibition, while orange color shows gene leads to activation, while the dotted line shows its indirect interaction. AR: androgen receptor

**Figure 3:**
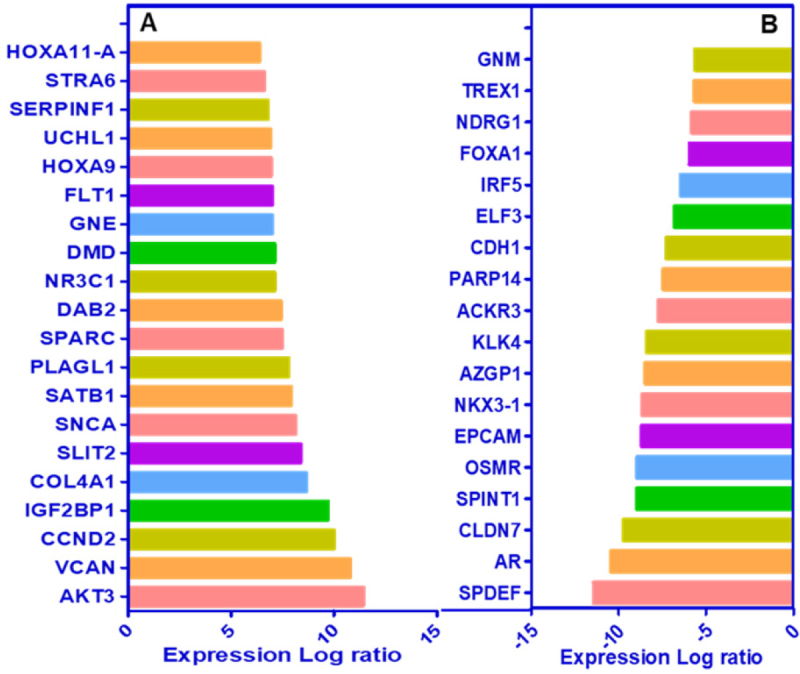
Cascade of upstream and downstream transcriptional regulators. The NGS data of LNCaP-enzalutamide resistant cells identified (A) upstream and (B) downstream regulators, based on overlap p-values computed based on significant overlap between genes in the dataset and known targets regulated by the transcriptional regulator and represented in graph on scale of expression log ratio at overlap *P*-value ≤ 0.001. NGS: next generation sequencing

Recent studies from our laboratory focused on the generation of antiandrogen resistant cell lines. For these studies, we treated androgen responsive human prostate cancer LNCaP cells with increasing concentrations of enzalutamide (1~20 μmol/L) by passage in media containing enzalutamide for six months. The resistant cells generated from 20 µmol/L enzalutamide were maintained in media containing 5 µmol/L enzalutamide and referred to as LNCaP-enzalutamide resistant cells. The LNCaP parental cells and LNCaP-enzalutamide resistant cells were subjected to RNA isolation followed by RNA-Seq analysis. The RNA-Seq data of LNCaP-enzalutamide resistant cells identified 4,578 upregulated and 4,184 downregulated transcripts. These differentially expressed genes were overrepresented by genes related to fatty acid oxidation, drug resistance signaling, drug metabolism, glucose and bile acid biosynthesis, lipid metabolism, fatty acid α/β oxidation, type II diabetes mellitus, and NF-κB signaling pathway. In contrast, signaling pathways such as cell cycle, Wnt signaling, and DNA repair pathways were downregulated. In particular, prolong suppression of AR by enzalutamide resulted in perturbations in the AR signaling pathway [Figure 2]. Analysis for the upstream regulators in enzalutamide resistant cells exhibited increased expression of transcription regulators (HOXA9, IGF2, SATB1, and PLAGL1), kinases (AKT3 and FLT1), peptidase (UCHL1), and ligand-dependent nuclear receptor NR3C1 [Figure 3A]. Moreover, a subset of genes that include transcriptional regulators such as *SPDEF*, *NKX3*.1, *ELF3*, *IRF5*, *FOXA1*, and G-protein coupled receptors were downregulated in enzalutamide resistant cells, compared to those of the parental LNCaP cells [Figure 3B].

The study provides information on some new lead molecules altered during enzalutamide resistance in prostate cancer.

## AR-dependent resistance mechanisms

Abiraterone acetate and enzalutamide are drugs frequently prescribed for advanced prostate cancer; however, at least 20%-40% of patients develop primary resistance. Ultimately, patients who exhibit clinical or biochemical responses to treatment with these agents eventually develop secondary resistance through complex pathways^[16]^. Development of resistance is mediated by the AR, which include AR amplification, AR overexpression, AR somatic point mutations, constitutively active AR splice variants, and altered intratumoral androgen biosynthesis.

Clinical evidence demonstrates that AR amplification is important in developing resistance to antiandrogens. A study by Mostaghel *et al*.^[31]^ demonstrated 3-fold increase in AR expression following treatment with abiraterone acetate in CRPC xenograft models. Another study that aims to probe the genomic landscape used liquid biopsies and circulating tumor DNA (ctDNA) obtained from prostate cancer patients and identified that AR amplification results in less responsive to antiandrogens. Approximately 50% of patients pretreated with either enzalutamide or orteronel (a CYP17A1 inhibitor) prior to abiraterone acetate treatment exhibited evidence of AR amplification, and only 13% of those with AR amplification demonstrated a response with > 50% PSA decline after being treated with abiraterone acetate. Approximately 5%-30% of mutations in AR were noted in circulating tumor cells and circulating tumor DNA of CRPC patients. Studies have shown that AR point mutations confer resistance to enzalutamide and abiraterone acetate^[32]^. The most frequently reported somatic mutation F877L/F876L has been identified in patients treated with enzalutamide and apalutamide. Binding to the mutated AR at ARN-509, these drugs act as agonists rather than antagonists^[33]^. Other mutations associated with poor response to enzalutamide are L702H, conferring acquired responsiveness to glucocorticoids, and T878A/T877A, resulting in progesterone-mediated activation of the AR. The W742C/L mutation is also reported to be responsible for the bicalutamide resistance^[32-34]^. Apart from the mutations in LBD, more than 30 mutations have been reported in the other parts of the receptor. Buchanan *et al*.^[35]^ identified somatic mutations within the N-terminal polyglutamine tract of AR. In the NTD of AR gene, there is a polymorphic trinucleotide repeat region (CAG)n which encodes for the polyglutamine tract in AR protein. In healthy individuals the number of these repeats ranges 6 to 39 and variation in the number of these repeats is found associated with onset of prostate cancer^[36-39]^. Interruption of the polyglutamine tract with two leucine residues (AR-polyQ2L) is reported to reduce the ligand induced N- and C-terminal interaction and result in the higher activity of receptor in comparison to wild type receptor. This high activity of AR receptor is attributed to the enhanced interaction with co-activators such as androgen receptor-associated protein 24^[35,40]^. There are evidences that suggest that enhanced interaction with co-activators strengthens the AR signaling in androgen depleted environment and in presence of weak AR agonist. This consequently results in the failure of antiandrogen therapy^[41]^.

Androgen receptor splice variants (AR-Vs) are also involved in the progression of prostate cancer, as well as the development of resistance to antiandrogens^[42]^. Prostate cancer progression occurs when AR-Vs lack the LBD and remain constitutively active, and their expression is amplified during ADT^[43,44]^. AR-V7 is one of the most widely studied splice variant in prostate cancer research. A study conducted by Chen *et al*.^[45]^ reported the high expression of AR-V7 in CRPC patients. Another study by Antonarakis *et al*.^[30]^ reported the high proportion of AR-V7 in circulating tumor cells and found it to be associated with the enzalutamide and abiraterone acetate resistance. Proteins encoded by AR-V7 lack the LBD domain of AR which is directly targeted by both enzalutamide and abiraterone acetate. Furthermore, AR-V7 proteins remain constitutively active in a ligand independent manner which gives AR-V7 expressing cells a selective advantage in an androgen depleted environment^[43]^. Another important AR-V reported in prostate cancer is ARv567e. It lacks exons 5, 6, and 7 which encode the LBD of AR. Interestingly, ARv567e is reported to induce oncogenesis autonomously^[46]^. While expression of AR-V7 is reported both in benign and malignant prostate cancer tissues, ARv567e expression has been associated with only malignant prostate cancer tissues^[46-48]^. Tagawa *et al*.^[49]^, with a cohort of 54 CRPC patients, reported the presence of AR-V7 splice variant in 36 patients (67%), ARv567e in 42 patients (78%), and the presence of both variants in 29 (54%) patients, but in 5 patients, both variants were absent. Previously, it was supposed that AR splice variants activate AR signaling independent of full-length AR, but it was found that ARv567e binds with full length AR to initiate ligand independent AR signaling which results in the cellular proliferation in the absence of androgen^[50]^. Apart from these two major AR-Vs other splice variants have been detected in CRPC such as AR-V1, AR-V2, AR-V3, AR-V4, AR-V5, AR-V6, AR-V8, and up to AR-V14^[51]^. AR-V1, AR-V4, and AR-V6 can dimerize with AR-V7 and AR-FL and therefore nuclear localization of these variants is induced by AR-V7 and AR-FL. Nuclear localization of these additional AR-Vs results in the enhanced activation of canonical AR targets and splice variant specific targets which consequently increases the severity of prostate cancer^[52]^. AR-8 is another important splice variant highly expressed in CRPC cell lines such as C4-2, C4-2B, and CWR22Rv1. AR-8 has been found truncated at C-terminal and it does not possess DBD or LBD this it lack of trans-activating function. AR-8 remains attached to the plasma membrane via its two palmitoylated cysteine residues and associates with AR-FL and EGFR to mediate Src-induced AR activation^[53]^.

Testosterone and 5α-dihydrotestosterone are sex steroid hormones that are largely synthesized from cholesterol in the testes and partially in the adrenal glands. Intratumoral synthesis of these hormones increases in CRPC patients. Cholesterol and weak androgens synthesized in the adrenal glands, such as androstenedione and dehydroepiandrosterone, may act as precursors of testosterone and 5α-dihydrotestosterone^[24]^. These augmented intratumoral levels of androgens promote both paracrine and autocrine activation of the AR regardless of systemic hormone levels. Studies conducted in cell culture and *in vivo* models suggests that enzalutamide resistance may be related to overexpression of genes encoding for enzymes involved in androgen biosynthesis such as SRD51A, HSD3β1, and AKR1C3^[54]^. One of these enzymes, AKR1C, is a potential target for pharmacological approaches against AR resistance. In both *in vitro* and *in vivo* preclinical models, indomethacin, an NSAID, inhibits AKR1C activity and re-sensitizes CRPC to enzalutamide.

The half-life of AR increases upon binding with the androgen with reduction in its degradation. Both the ubiquitin-proteasome system and the autophagy-lysosome pathway play important roles in the degradation of AR protein. Reports suggest several E3 ubiquitin ligases ubiquitinate AR proteins with distinct AR domain interactions including UBE3A (E6-AP), RCHY1 (ARNIP), and CHIP binding to the AR N-terminal domain, RNF4 (SNURF) to the AR DNA-binding domain while RNF6 and SIAH2 bind to the AR ligand binding domain^[55-60]^. In addition, Ser-213 and Ser-791 phosphorylation is essential for AR degradation by MDM2 E3 ligase while Thr-850 phosphorylation may stabilize AR by recruiting RNF6. AR phosphorylation at Ser-578 by p21-activated kinase 6 (PAK6) promotes the association between AR and MDM2 to activate MDM2-mediated proteasomal degradation of AR^[47]^. Reports suggest that RNF6 and SIAH2 may represent effective therapeutic targets. In contrast, proteases such as ubiquitin-specific peptidases or deubiquitinases hydrolytically cleave ubiquitin or ubiquitin-like proteins from their substrates affect AR transcriptional activity and stability^[61]^. USP7, USP10, USP14, and USP26 are some deubiquitinases which affect AR stability and its binding and are thus important therapeutic targets to control CRPC progression^[62]^.

## AR-independent resistance mechanisms

### PI3K-Akt signaling

The PI3K-Akt-mTOR pathways are well known to regulate all major cellular processes such as cell growth, development, proliferation, protein synthesis regulation, and programmed cell death^[63]^. Aberrant activation of PI3K-Akt has been implicated in prostate cancer development and progression^[64]^ and activation of this pathway may be responsible for antiandrogen drug resistance^[33]^. Activation of PI3K signaling and loss of the tumor suppressor gene *PTEN*, the negative regulator of PI3K/Akt pathway, occurs prominently in metastatic prostate cancer^[65,66]^. A phase II randomized clinical trial, those having *PTEN* loss demonstrated the trends for improvement with the addition of Akt inhibitor to abiraterone acetate/prednisone treatment^[67]^. Studies on preclinical models have shown that the activation of PI3K/Akt pathway is critical to CRPC development^[68]^. PI3K isoform, especially p110α, is associated with the insulin and growth factor pathways while p110β isoform is known to regulate cell mitosis and survival^[69]^. Overexpression of p110β is specifically involved in prostate cancer growth and proliferation^[70]^. Interaction of AR with Src kinase and p58α regulatory subunit of PI3K activates mitogen-activated protein kinase (MAPK) and Akt pathways, leading to increased cell proliferation and survival^[71]^. In addition, activation of MAPK and Akt also enhance AR signals by phosphorylating the AR or transcriptional co-activators^[65]^. During the castration stage, Akt directly phosphorylates AR at two locations on Ser-791 and Ser-217; however, its clinical significance is not been established. Chandrasekar *et al*.^[72]^ demonstrated higher level of Src activation (androgen-independent) was associated with worse prostate cancer phenotypes like unlimited cell growth, tumor migration and invasion, and inhibition of apoptotic pathways. Besides, several clinical trials are being conducted via combination therapy of molecular targeted drugs like tyrosine kinase inhibitors with second-generation antiandrogens drugs. Thomas *et al.*^[73]^ in an *in vivo* study showed synergistic targeting of the PI3K/Akt pathway, and they showed that the AR axis significantly delayed CRPC progression.

### Glucocorticoid signaling

Glucocorticoid receptor (GR), a member of the steroid hormone nuclear receptors family whose structure and mechanism of action is similar to androgen receptor, is expressed in almost all human tissues^[74]^. GRs complexed with heat shock proteins are found in the cytoplasm. GRs consist of four functional domains, similar to ARs, including DNA binding domain, ligand binding domain, N-terminal, and the hinge region^[75]^. ARs and GRs share similar transcriptomes and response elements in target genes. The binding of glucocorticoids promotes homodimerization and translocation in the nucleus where GR mediates transcriptional activation and influences target gene expression^[76]^. The role of glucocorticoids and GRs in prostate cancer is complex as it influences both harmful and beneficial effects. Venkitaraman *et al.*^[77]^ demonstrated that glucocorticoids inhibit lymphangiogenesis through vascular endothelial growth factor downregulation and inhibit prostate cancer cell proliferation through the glucocorticoid receptor induction. Induction of GR upregulates p21 and p27 expression and downregulate oncogenic molecules such as MAPKs, NF-κt, and STAT1^[78]^. GR mediates a similar but distinct set of AR-target gene expression. Glucocorticoids initially have suppressive effects on prostate cancer and are often given in conjunction with early treatments of CRPC with chemotherapy and second-generation antiandrogen agents (enzalutamide or abiraterone acetate)^[72]^. Both AR and GR possess similar DNA binding domain structures. GR has been shown to share response elements with many AR regulated target genes which ultimately leads to its upregulation in patients treated with chemotherapy along with second-generation antiandrogens^[79]^. Upregulated GR signaling mediates resistance to androgen-targeted agents and the mechanism is known as “glucocorticoids receptor take-over” pathway^[80]^. A recent investigation by Puhr *et al*.^[81]^ on GR expression and its functional significance in both prostate cancer cell lines and prostate cancer patients found that GR expression is low in primary prostate cancer tissue but is significantly increased during long-term exposure to enzalutamide. Similar findings were reported by Arora *et al.*^[79]^ who demonstrated how GR overexpression conferred resistance to enzalutamide. Li *et al*.^[82]^ further determined that treatment with enzalutamide maintained the cortisol level and enhanced glucocorticoid signaling by inhibiting the 11β-hydroxysteroid dehydrogenase-2 (11β-HSD2).

### NF-κB signaling

The NF-κB family proteins are important component of the oncogenic pathway in multiple human malignancies^[83]^. There are five distinct NF-κB proteins of which p65/p50 heterodimer has shown to be constitutively active in prostate cancer^[84]^. Moreover, activation of NF-κB/p52 pathway has been implicated in the development of resistance to prostate cancer^[85]^. Overexpression of p52 increases glucose uptake and produces higher ATP and lactate levels moderating enzalutamide resistant in CRPC cells^[86]^. It has been shown that processing of p100 to p52 through molecules such as B-cell activating factor, CD40, lymphotoxin β, and STAT3 may activate AR-Vs and glucose metabolism frequently involved in CRPC progression^[87]^. Activation of AR-Vs mediated by heterogeneous nuclear RNA-binding protein (hnRPA-1) leads to significant hyperplasia and induced castration resistance growth^[88]^. This leads to inhibition of apoptosis and cell cycle and thus limiting sensitivity to second-generation antiandrogen therapy. Liu *et al*.^[18]^ showed that overexpression of androgen receptor splice variant-7 (AR-V7) could activate NF-κB which in turn upregulates interleukin IL-6 gene expression. The study indicates a positive interaction between AR-V7 expression and activated NF-κB/IL-6 signaling in CRPC pathogenesis. They also demonstrated that AR-V7-induced NF-κB activation and IL-6 gene transcription could be inhibited by melatonin in LNCaP and 22Rv1 cells. Nadiminty *et al*.^[89]^ demonstrated that the mechanism of resistance to enzalutamide to be mediated by AR splice variants that lack the ligand binding domain (LBD); the AR variants expression are increased by NF-κB signaling. This group further demonstrated that downregulation of NF-κB2/p52 expression in CRPC cells by short hairpin RNA abrogates splice variants expression^[89]^. Downregulation of NF-κB inhibits AR-Vs expression and restores the sensitivity of CRPC to second-generation antiandrogen therapy and desensitizes cells to androgens^[90]^. Another study reported that downregulation of hnRPA-1 may lead to the re-sensitization of enzalutamide resistant prostate cancers^[91]^. NF-κB has been shown to regulate the expression of several cytokines, in particular IL-6, in normal tissues and cancer cells. IL-6 is highly expressed in CRPC, increasing the transcriptional activity of the AR in a ligand-independent manner^[92]^. But experimental therapies against IL-6 and the clinical trials on patients with late-stage prostate cancer has not been yet reported. IL-6 acts through the Janus kinase (JAK)-signal transducer and activator of transcription (STAT) pathway which has been approached as anti-STAT3 therapeutics in several human cancers including prostate cancer^[93]^. Treatment of prostate cancer cells with the JAK inhibitor AG490 leads to the re-sensitization of cells to enzalutamide^[94]^. Liu *et al.*^[94]^ demonstrated that co-treatment with enzalutamide and AG490 has an inhibitory effect on cell growth and induces apoptosis. Another study demonstrated that combinational therapy of antiandrogens with NF-κB inhibitors efficiently inhibits tumor growth of human CRPC xenografts.

### FOXO signaling

Studies reveal that members of FOXO family also develops antiandrogen resistance. Many pro-apoptotic proteins such as caspase-9, Bad, and FOXO subfamily members including FOXO1, FOXO3a, and FOXO4 are phosphorylated by Akt to maintain cell survival^[95-98]^. The phosphorylated form of FOXO proteins remains inactive in the cytoplasm. Dephosphorylation of these proteins occurs due to Akt activity inhibition by several cellular factors such as PTEN or PI3K inhibitors. Activated FOXO proteins translocate from the cytoplasm to the nucleus and subsequently bind to promoters of their target genes such as p27, FASL, Bim-1, p21, Cyclins (A, B, D, E, and G2), PGC1α, RAG1, RAG2, *etc*.^[99]^. FOXO3a increases androgen receptor expression by direct binding to the AR gene promoter^[100]^. However, FOXO1 decreases AR transactivation by engagement of histone deacetylase HDAC3 proteins^[101]^. Inactivated FOXO proteins have been implicated in prostate cancer progression towards castration resistance. Das *et al.*^[102]^ demonstrated that activation of Akt induces FOXO3a inactivation, meaning that PI3K/Akt inhibitors would activate FOXO3a. Ketola *et al*.^[103]^ highlighted the role of FOXM1 as the key player of the most aggressive prostate cancer subtype 1 (PCS1) of CRPC. Akt pathway upregulates the FOXM1, making it a target of Akt inhibitors^[104]^. FOXO1 also controls cell survival in hepatic cells via gluconeogenesis modulation by cooperating with PGC-1α^[105]^.

### WNT signaling

Genome-wide analysis of prostate cancer has found that aberrant Wnt/β-catenin signaling drives the metastatic growth of prostate cancer^[106]^. Wnt signaling constitutes both canonical (β-catenin dependent) and non-canonical (β-catenin independent) signaling pathways that regulate cell fate, proliferation, differentiation, migration, and self-renewal^[107]^. Some studies demonstrated that AR and Wnt/β-catenin pathways intersect each other^[106]^. It has been reported that expression of β-catenin and AR protein shows significant correlation in CRPC tumors^[108,109]^. AR and β-catenin may together lead to the expression of target genes promoting androgen-independent growth and progression^[110]^. Aberrant Wnt signaling is involved against ADT resistance after and prior to antiandrogen therapy^[111,112]^ and elevated level of β-catenin has been found in post-ADT specimens^[113]^. CRPC patients more frequently develop genetic changes compared to treatment-naïve prostate cancer^[114]^. Gain and loss of functional mutation in the β-catenin gene (CTNNB1) and APC encoding gene alter the Wnt signaling in CRPC^[12,115]^. Wnt-pathway activating mutations have been associated with resistance to antiandrogens in CRPC tumors^[116]^. Nuclear localization and cytoplasmic accumulation of β-catenin and abnormal β-catenin expression has been observed in the specimens obtained from CRPC patients^[117,118]^. Chen *et al.*^[119]^ demonstrated that suppression of non-canonical Wnt pathway overcomes enzalutamide resistance in CRPC. A study reported that enzalutamide-resistant cells show upregulation of β-catenin and AR which may be partially due to the reduction of β-TrCP mediated-ubiquitination. The study also demonstrates the correlation between AR and β-catenin playing a critical role not only in prostate cancer initiation but also in chemotherapy resistance progression^[120]^. In various models, synergistic combination of β-catenin inhibitor (ICG001) with enzalutamide inhibit cell proliferation, tumor growth, and stem-like markers expression. However, the role of Wnt/βcatenin signaling in promoting enzalutamide resistance in prostate cancer has not yet been reported. Reports suggests that cellular signaling pathways like PI3K-Akt, Wnt, glucocorticoid, NF-κB, FOXO, and in others like ONECUT2 are involved in second-generation antiandrogen resistance in AR-independent pathways.

### Cytokine signaling

The Janus kinase and signal transducer and activator of transcription (STAT) 3, and its downstream effector IL-6 lead to AR activation^[121]^. Human and murine prostate cancer models have exhibited varying effects of IL-6 and/or STAT3 on tumor cell growth. Experimental treatments have been proposed that block the IL-6/STAT3 signaling pathway. The anti-IL-6 antibody siltuximab (CNTO 328) has been shown to delay development of castrate resistance in prostate cancer *in vitro* and *in vivo*^[122]^. However, the anti-IL-6 antibody has not been validated clinically as a monotherapy in phase II clinical trials^[121]^. IL-6 is postulated to be involved in regulation of cellular stemness via phosphorylation of STAT3 and it is also thought to play a role in the development of resistance to enzalutamide^[122]^. Endogenous inhibitors of IL-6 suppress cytokine signaling and inhibit activated STAT. While they inhibit signal transduction through STAT3, they may also exert anti-apoptotic effects^[122]^. Given the complexity of IL-6 interactions in prostate cancer, a customized approach is required to identify patients who will benefit from anti-IL-6 therapy in conjunction with standard treatments.

## Other resistance mechanisms

### Autophagy

The other mechanism by which CRPC develops resistance to enzalutamide treatment includes autophagy. Autophagy is an adaptive^[33]^ and catabolic process to maintain cellular homeostasis through degradation and recycling of cellular components^[123]^. It is constitutively active at a low basal rate and is activated in response to stressors. This process allows cells to degrade cellular proteins and organelles through lysosomes to generate energy^[124]^. Also, the physiological balance between autophagy and apoptosis are supposed to be lost in cancer^[125]^. Conditions such as metabolic stress and hypoxia leads to consequent upregulation of autophagy which forms a good source of nourishment for highly proliferative tumor cells^[126]^. Androgen deprivation has shown to induce autophagy, but the exact mechanism remains unknown. An RB1 study demonstrated that the autophagy inhibitors clomipramine and metformin significantly increased the cytotoxicity of enzalutamide *in vitro*^[127]^. An *in vivo* study on enzalutamide/clomipramine found that the drug combination reduced prostate tumor size by 91% as compared to enzalutamide/metformin combination which reduced the tumor size by 78%^[127]^. Heat shock chaperone like protein-clusterin is also known to mediate autophagy induced by AR antagonists such as enzalutamide^[33]^. Synergistic administration of enzalutamide and clusterin inhibitor (OXG-011) enhanced apoptosis and delayed progression in both *in vitro* and *in vivo* prostate cancer models^[128]^.

### Epithelial-mesenchymal transition

The epithelial-to-mesenchymal transition (EMT) is induced by ADT in metastatic prostate cancer, and studies demonstrate that this process promotes tumor progression and drug resistance^[33]^. Activation of pathways such as TGF-β and SMAD alters the activity of some transcriptional factors such as Snail and Twist, reducing the expression of E-cadherin, a key event in EMT^[129]^. Post-enzalutamide treatment and ADT result in upregulation of Twist and this alteration, together with protein kinase C (PKC) activation, facilitates drug resistance. A study found that using a combination of Ro318220 (PKC inhibitor) with enzalutamide altered resistance in prostate cancer^[130]^. Snail, another transcriptional factor, can promote resistance to enzalutamide and increase migration and invasion of prostate cancer cells^[131]^.

### Neuroendocrine differentiation

Neuroendocrine prostate cancer (NEPC) is an aggressive form of prostate cancer that may arise in patients who underwent treatment like hormonal therapies for prostate adenocarcinoma^[132]^. It is characterized by the loss of AR signaling during neuroendocrine trans-differentiation which results in resistance to enzalutamide therapy^[133]^. Reports suggest that 30% of metastatic CRPC belongs to NEPC and 1% of primary prostate cancers are diagnosed as NEPC^[134]^. Several reports suggest that N-Myc is a driver of NEPC. Genomic amplification and overexpression of N-Myc and Aurora kinase A are also associated with differentiation of prostate cancer to NEPC^[135]^. A preclinical study reported that overexpression of N-Myc abrogated AR signaling and developed features similar to NEPC^[136]^. Knockdown or inhibition of Aurora kinase A with inhibitor alisertib (formerly MLN8237) resulted in decreased N-Myc target gene expression and cell viability and destabilizes N-Myc protein levels^[136,137]^. In another preclinical study, CD532, another Aurora kinase A inhibitor, was shown to reduce N-Myc protein levels indicating viable treatment options for patients with NEPC^[137]^. Guo *et al*.^[138]^ demonstrated that ectopic expression of ONECUT2 (transcription factor) in prostate adenocarcinoma in combination with hypoxia suppresses androgen signaling. ONECUT2 drives neuroendocrine prostate cancer by regulating hypoxia signaling. It activates SMAD3 which regulates hypoxia signaling through modulating HIF-1α chromatin-binding^[139]^. It is also reported that treatment with hypoxia-activated prodrug TH-302 potentially reduces NEPC tumor growth. A study reported that both tumor suppressor proteins TP53 and RB1 are important factors in NEPC differentiation^[140]^. Another study reported that TP53 and RB1 androgen-dependent prostate cancer shift to androgen-independent NEPC after enzalutamide treatment^[16]^. Loss of TP53 and RB1 and the phenotypic switch of prostate cancer is mediated by the expression of a transcription factor SOX2. Mu *et al.*^[141]^ showed that inhibition of SOX2 restored TP53 and RB1 function. Tan *et al*.^[142]^ observed that approximately 90% of NEPC cases was associated with loss of RB1 and 85% of cases with RB1 deletions. It has been suggested that another factor involved in the development of NEPC is the enhancer of zeste homolog 2 (EZH2), an epigenetic reprogramming gene which represses androgen and drives neuroendocrine prostate cancer^[143]^. In preclinical studies, various EZH2 inhibitors like GSK-126/343/503 that targets EZH2 enzyme activity demonstrated growth inhibition in prostate cancer cell lines^[144]^. Ku *et al*.^[144]^’s study on double knockout mice demonstrated that blocking of EZH2 may re-sensitize to enzalutamide PTEN and RB.

### Immune resistance

Several studies demonstrated that PD-L1 is considerably expressed in enzalutamide-resistant cell lines and enzalutamide-resistant tumors in animal models^[145]^. These results suggest that CRPC progression and resistance to AR signaling pathways is facilitated by PD-L1 and PD-1^[33]^. Mechanisms of primary resistance can be further elucidated through phase II clinical trials investigating the role of immunotherapy in CRPC, involving treatment with ipilimumab in combination with abiraterone acetate in treatment-naïve CRPC (NCT01848067). The second phase II clinical trial is currently evaluating combination immune checkpoint blockade with ipilimumab and the PD-1 inhibitor nivolumab in mCRPC patients positive for AR-V7 (NCT02601014).

### TMPRSS2-ERG fusion

TMPRSS2-ERG gene fusion (T-E fusion) is the most common fusion event associated with CRPC. Two different studies reported the presence of T-E fusion in 9 out of 15 and 14 out of 19 CRPC tumor samples^[22,146]^. Moreover, tumor samples containing T-E gene fusion were reportedly more aggressive in comparison to T-E fusion negative samples, suggesting distinct molecular and perhaps clinical subtypes^[147]^. The first gene fusion in prostate cancer was reported was TMPRSS2-ETV1. Later on other fusion transcripts (TMPRSS2-ETV4 and TMPRSS2-ETV5) involving TMPRSS2 and ERG proteins have also been reported^[148]^. Different mechanisms are reported to be involved in the formation of these fusion transcripts. For example, the fusions of TMPRSS2-ERG and TMPRSS2-ETV4 are caused by intra-chromosomal deletion and fusion or intra-chromosomal translocation^[147]^. In CRPC, interstitial deletions are the most common cause of T-E fusion. This high frequency of T-E fusion at this part of chromosome 21 suggests that it is a hotspot for chromosomal rearrangements. T-E fusion results in androgen dependent upregulation of ERG as one of the most common genomic dysregulation in prostate cancer. Some studies have reported that improved response to abiraterone acetate is associated with the ERG gene rearrangement. ERG is also reported to guide the expression of steroidogenic enzyme AKR1C3, highly expressed in enzalutamide resistant cells^[149]^. Further investigation revealed the presence of the ERG/AKR1C3/AR feed-forward loop which confers androgen synthesis, AR signaling, and resistance to AR targeting agents in CRPC^[150]^. Estrogen receptor (ERα) is reported to upregulate the expression T-E fusion transcript. Based on this evidence, it can be postulated that ERα antagonists might offer better therapeutic outcome by lowering the expression level of T-E fusion transcripts. Attard *et al*.^[151]^ tested this hypothesis using abiraterone acetate and results showed that five out of six patients responded to this therapy. HDACs inhibitors are reported to inhibit genes involved in the formation T-E fusion transcript.

## Conclusion and future prespectives

**Figure 4:**
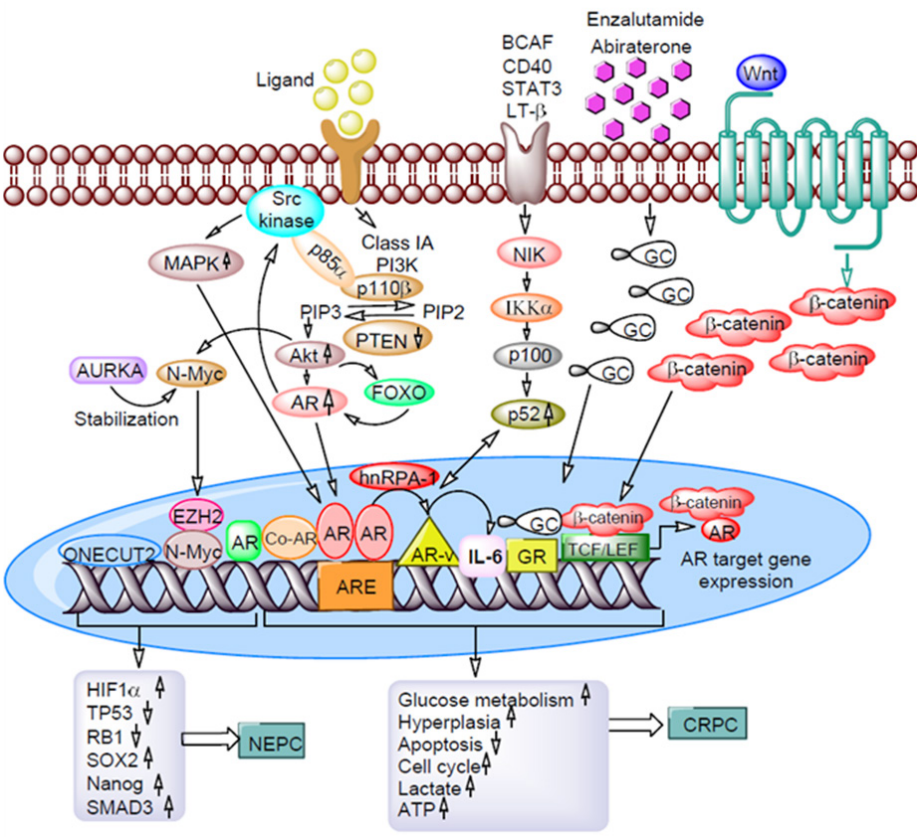
Mechanisms of enzalutamide and abiraterone acetate resistance in prostate cancer cells. Aberrant activation of PI3K/Akt pathway overexpressing p110β with loss of *PTEN* gene. Interaction of p85α subunits with Src kinase activates MAPK. Activated Akt overexpresses N-Myc, FOXO, ONECUT2 and EZH2 leads to activation of HIF1α, SMAD3, SOX2, and Nanog through suppressing TP53 and RB1. AR in the presence of hnRPA-1 forms AR-Vs and activates NF-κB signaling which in turn up-regulates IL-6 gene expression. Long-term exposure to antiandrogens significantly increases GR expression. Interaction of aberrant β-catenin of Wnt signaling to AR leads to expression target genes involved prostate cancer cell proliferation, tumor growth, stem cell marker expression, and chemotherapy drug resistance. Interaction of these genes with each other and with AR promotes enzalutamide/abiraterone acetate mediated neuroendocrine prostate cancer (NEPC) and castration resistant prostate cancer (CRPC) formation. PI3K: phosphatidylinositol-3 kinase; PTEN: phosphatase and tensin homolog; MAPK: mitogen-activated protein kinase; EZH2: enhancer of zeste homolog 2; HIF1α: hypoxia-inducible factors- α; SOX2: SRY-box transcription factor 2; TP53: tumor protein 53; RB1: retinoblastoma1; hnRPA-1: heterogeneous nuclear ribonucleoprotein A1; IL-6: interleukin-6; AR-vs: Androgen receptor variants; AR: androgen receptor; GR: glucocorticoid receptor; CRPC: castration resistant prostate cancer; NEPC: neuroendocrine prostate cancer; BCAF: B-cell activating factor; LT: Lymphotoxin-β; AURKA: Aurora kinase A

The advent of new generation hormonal therapies set the stage for a new era in the treatment landscape of CRPC. Although these agents showed initial treatment benefit, a relevant proportion of patients do not benefit at all or acquire resistance during treatment. Increasing knowledge of the subject has provided information that prostate cancer is a heterogeneous disease with the coexistence of both AR-responsive and AR-refractory cancer cells responsible for antiandrogen resistance at various degrees. This heterogeneity might be a critical factor for different biological behaviors and particularly for different responses to new-generation hormonal therapies among various prostate cancer. The current mechanisms of enzalutamide resistance, the mechanisms and pathways involved to design approaches for overcoming resistance, and the problems and solutions associated with these mechanisms are summarized in Figure 4.

Intense ongoing research is needed to discern pathways and mechanisms to improve drug sensitivity. The more complete understanding of these mechanisms of resistance through approaches like next generation sequencing, single cell sequence, proteomics, and others will enable the development of improved treatment strategies to overcome this resistance. Moreover, development and validation of assays in identifying mechanisms of resistance and their clinical implementation will be useful in providing relevant predictive biomarkers and will become essential tools assisting clinicians for personalized treatment. Improving our understanding of these AR resistance mechanisms and translating them into the next generation of AR targeting agents will be key to designing more effective therapies for advanced-stage prostate cancer.

## Declarations

### Copyright

© The Author(s) 2020.
